# Supplementary material for: Causal Relationship between Aging and Anorexia Nervosa: A White-Matter-Microstructure-Mediated Mendelian Randomization Analysis
Source: Biomedicines. 2024 Aug 16;12(8):1874. doi: 10.3390/biomedicines12081874 (PMC11351342; doi:10.3390/biomedicines12081874)
Supplement: Supplementary file 1 [file biomedicines-12-01874-s001.zip › Supplementary Figure/Figure S8/Leaveoneout plot of MR analysis for TL to ubm-b-1969.pdf]

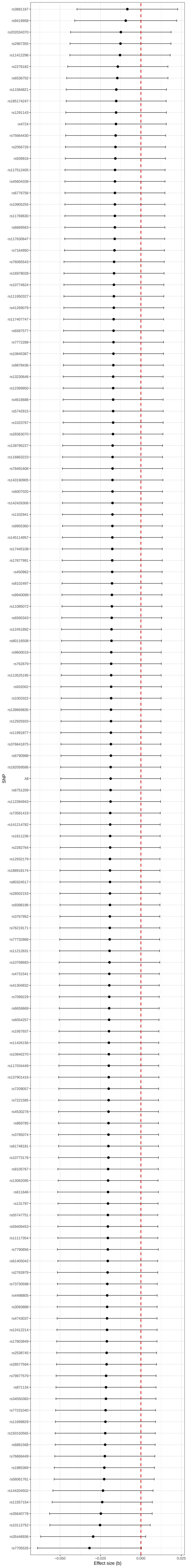
rs3891167
rs9419958
rs202034370
rs2967355
rs11412296
rs2276182
rs6536702
rs11584821
rs185174247
rs1291143
rs4724
rs75664430
rs2056726
rs939916
rs117512405
rs45604339
rs6776756
rs10905255
rs11769630
rs6669563
rs117630647
rs7164950
rs76065543
rs16978028
rs10774624
rs111950327
rs41269079
rs117407747
rs6587577
rs7772289
rs10845387
rs9878436
rs13230646
rs12369950
rs4616688
rs5742915
rs1023767
rs28363070
rs139795227
rs116863223
rs78491606
rs143190905
rs6007020
rs142426306
rs1332941
rs9955360
rs145114957
rs17445108
rs17677991
rs450962
rs8102497
rs9940099
rs11085072
rs6590343
rs12451892
rs80116508
rs9600019
rs762679
rs113525195
rs932002
rs1003322
rs139669835
rs12925933
rs11991877
rs376641875
rs6790988
rs182059586
All
rs6751209
rs112394943
rs73581419
rs141214782
rs1611236
rs2282764
rs12932179
rs188918174
rs80324517
rs28502153
rs9398196
rs3767952
rs76219171
rs77732866
rs11212631
rs10768683
rs4731541
rs41304832
rs7099229
rs6659669
rs6054257
rs1957937
rs11426156
rs10840270
rs117034449
rs137901416
rs7209057
rs7221585
rs4530278
rs869785
rs3785074
rs61748181
rs10773176
rs8105767
rs13062095
rs611646
rs131797
rs55747751
rs59409453
rs11117354
rs7790856
rs61405042
rs2763979
rs73730598
rs4498805
rs3093888
rs4743037
rs12412214
rs17803849
rs2538745
rs28577594
rs79977579
rs871134
rs34550383
rs77231040
rs11699829
rs150150565
rs6881568
rs76666449
rs1985369
rs56061761
rs144204502
rs11557154
rs35640778
rs10112752
rs35446936
rs7705526
SNP
-0.050
-0.025
0.000
0.025
Effect size (b)
